# Distribution of bovine and rabbit lens α-crystallin products by MALDI imaging mass spectrometry

**Published:** 2008-01-29

**Authors:** Angus C. Grey, Kevin L. Schey

**Affiliations:** Department of Cell and Molecular Pharmacology, Medical University of South Carolina, Charleston, SC

## Abstract

**Purpose:**

To develop a general tissue preparation protocol for MALDI (Matrix-Assisted Laser Desorption Ionization) imaging mass spectrometry of ocular lens tissue, and to compare the spatial distributions of α-crystallin and its modified forms in bovine and rabbit lenses.

**Methods:**

Frozen bovine and rabbit lenses were cryosectioned equatorially at −20 °C into 12 μm-thick tissue sections. Lens sections were mounted onto conductive glass slides by ethanol soft-landing to maintain tissue integrity. An ethanol/xylene washing procedure was applied to each section before matrix application to facilitate uniform matrix crystal formation across the entire tissue section. Molecular images of both α-crystallin subunits and their modified forms were obtained from mass spectral data acquired at 100 μm steps across both whole rabbit and half bovine lens sections.

**Results:**

Distinct spatial patterns for the two subunits of α-crystallin and their modified forms were observed in the rabbit and bovine lens sections. While αA-crystallin was extensively degraded in the lens core of both species, rabbit lenses exhibited a greater degree of larger molecular weight truncation products. In contrast, αB-crystallin degradation was limited in both species. Interestingly, phosphorylation of αA- and αB-crystallin was most abundant in the middle cortex of both species.

**Conclusions:**

An improved method for investigating the spatial distribution of α-crystallin in the ocular lens by MALDI imaging mass spectrometry has been developed. The localization of multiple degradation products and specific regions of α-crystallin phosphorylation in bovine and rabbit lenses gives new insight into the program of lens fiber cell differentiation and normal lens function.

## Introduction

The mammalian lens is an avascular, transparent optical element that focuses incident light onto the retina. The bulk of the lens consists of highly elongated secondary lens fiber cells, which differentiate at the lens equatorial region from an epithelial cell monolayer that covers the lens anterior surface. Throughout this process, fiber cells develop several specializations that help maintain global lens homeostasis and, therefore, transparency. Among the most important specializations are the degradation of cell nuclei and other cell organelles to maintain a path free from light-scattering elements [1,2], the acquisition of junctional specializations, which maintain the extracellular space smaller than the wavelength of light [3], and the abundant expression of soluble lens crystallin proteins [4], which contributes to an increasing gradient of protein concentration and refractive index toward the center of the lens that aids visual acuity [5].

As a consequence of programmed lens cell differentiation, mature fiber cells lack the ability to synthesize new protein. Instead, posttranslational modification of existing proteins has been proposed as a mechanism for functional adaptation to a changing cell environment in differentiated lens fibers [6]. A variety of proteins, including connexins [7], aquaporin-0 [8], and crystallin proteins [9] have now been observed to alter function in differentiated fiber cells in response to common posttranslational modifications such as truncation and phosphorylation. The most abundant lens crystallin protein, α-crystallin, is primarily found as large molecular weight aggregates of two subunits, αA- and αB-crystallin. A member of the small heat shock protein family, α-crystallin is a molecular chaperone in the lens, acting to prevent nonspecific aggregation of denatured proteins [10]. The chaperone activity of α-crystallin is altered by truncation [11-13] and phosphorylation [9,14-18]. Therefore, information on the spatial distribution of α-crystallin and its modified forms is useful in understanding how the normal lens maintains transparency.

While immunolabeling can be used to investigate such distribution patterns, its ability to simultaneously detect more than three to four specific antibody probes is limited principally due to the small number of discrete recording channels available. Furthermore, the simultaneous detection of multiple truncation products of a single protein using immunolabeling is not possible for the simple reason that any antibody epitope will be present in more than one truncation product of any individual protein. In addition, detection of phosphoproteins using immunolabeling requires prior knowledge of phosphorylated residues in the protein of interest and rigorous validation of their antibody specificity.

Alternatively, the spatial distribution of unmodified and modified proteins can be investigated using two-dimensional gel electrophoresis of microdissected tissue regions. Posttranslational modification of human αA-crystallin was investigated using this method [19]. Increased truncation and modification of αA-crystallin correlated with lens fiber cell age and depth within the lens. Spatial resolution in this study was limited to two microdissected regions, although a protocol for human lens microdissection into six distinct regions has been reported [20]. Furthermore, the precise truncation sites and modifications could not be determined, and low molecular weight truncation products of αA-crystallin were not detectable using the electrophoresis method.

A recent report employed a new technology, MALDI (Matrix-Assisted Laser Desorption Ionization) tissue imaging, to simultaneously map the unmodified and numerous modified forms of α-crystallin [21]. In this study, extensive degradation of bovine αA-crystallin and phosphorylation of both α-crystallin subunits was detected. While α-crystallin phosphorylation appeared to be most abundant in the lens middle cortex, the MALDI images were not resolved enough to confirm this. Here, we present an improved tissue preparation method for obtaining high quality MALDI images of the ocular lens. The general applicability of the method is demonstrated in bovine and rabbit lenses where α-crystallin distributions are compared and contrasted. In addition, we use higher spatial resolution MALDI imaging techniques to confirm the presence of abundant phosphorylation of α-crystallin in the lens middle cortex of both bovine and rabbit lenses. This information adds to our growing understanding of protein modification that occurs as a function of lens fiber cell differentiation and age in the normal lens.

## Methods

### Reagents

Acetonitrile, high-performance liquid chromatography (HPLC)-grade water, formic acid, and sinapinic acid (SA) were purchased from Sigma-Aldrich (St Louis, MO). Anhydrous ethanol was purchased from Electron Microscopy Sciences (Hatfield, PA). ITO (Indium Tin Oxide) -coated conductive glass microscope slides were purchased from Bruker Daltonics (Billerica, MA). Frozen mature bovine lenses (approximately two years old) were obtained from Pel-Freez Biologicals (Rogers, AR). Rabbit eyes (approximately two years old) were obtained fresh from Pel-Freez Biologicals (Rogers, AR). The lenses were removed and stored at −80 °C until further use.

### Tissue preparation

Frozen bovine and rabbit lenses were attached to cold specimen chucks with the application of a small amount of optimal cutting temperature (OCT) embedding medium at the base of the tissue. Lenses were sectioned equatorially at −20 °C into 12 μm-thick tissue sections using a disposable blade stage-equipped cryostat (Microm HM 550, Walldorf, Germany). To collect frozen sections, a thin, uniform layer of anhydrous ethanol (room temperature, r.t.) was applied to the conductive glass slides (r.t.), and cryosections were thaw mounted by touching the glass slide to the tissue section. After drying, tissue sections were sprayed with several cycles of acetonitrile-water solution (50:50, vol/vol) with a TLC sprayer, resulting in a tightly bound section. After drying, tissue sections were washed successively for 60 s each in 70% ethanol, 95% ethanol, 100% ethanol, and xylene to facilitate uniform matrix crystal formation across the entire tissue section.

### Matrix deposition

A solution of 15 mg/ml SA freshly prepared in acetonitrile-water-formic acid (50:40:10, vol/vol/vol) was sprayed evenly onto the tissue sections using a TLC sprayer. Repeated cycles of matrix solution spraying were applied when tissue sections appeared mostly dry, approximately 45–60 s after previous spray application. Typically, a total volume of 15 ml was used to obtain an even coating with good quality test MALDI mass spectra.

### MALDI imaging

Mass spectrometric analyses were performed in the linear, positive mode at +20 kV accelerating potential on a time of flight mass spectrometer (Bruker Autoflex III TOF/TOF; Bruker Daltonik, Bremen, Germany), which was equipped with a Smartbeam laser capable of operating at a repetition rate of 200 Hz with optimized delayed extraction time. The laser beam size was set to small, estimated to be approximately 30 μm in diameter. Using Bruker Protein Standard 1 (Bruker Daltonik, Bremen, Germany), a linear, external calibration was applied to the instrument before data collection. Mass spectral data sets were acquired over approximately half of a bovine lens and a whole rabbit lens using flexImaging^TM^ software (Bruker Daltonik, Bremen, Germany) in the mass range of *m/z* 3,000-30,000 with a raster step size of 100 μm and 250 laser shots per spectrum. After data acquisition, molecular images were reconstituted using flexImaging^TM^ software. Each data set consists of approximately 12,000 individual sampling locations, each representing one pixel in the resultant image. Data was normalized to total ion current in each spectrum, and each *m/z* signal plotted ±8 mass units. For display purposes, data was interpolated and pixel intensities were re-scaled in flexImaging^TM^ to utilize the entire dynamic range. Assignments of protein identifications to *m/z* signals were made based on previous analysis of bovine lens crystallins [21] by matching observed *m/z* to predicted molecular weights of abundant lens crystallin proteins.

## Results

### MALDI MS spectra obtained from different regions of bovine and rabbit lens

**Figure 1:**
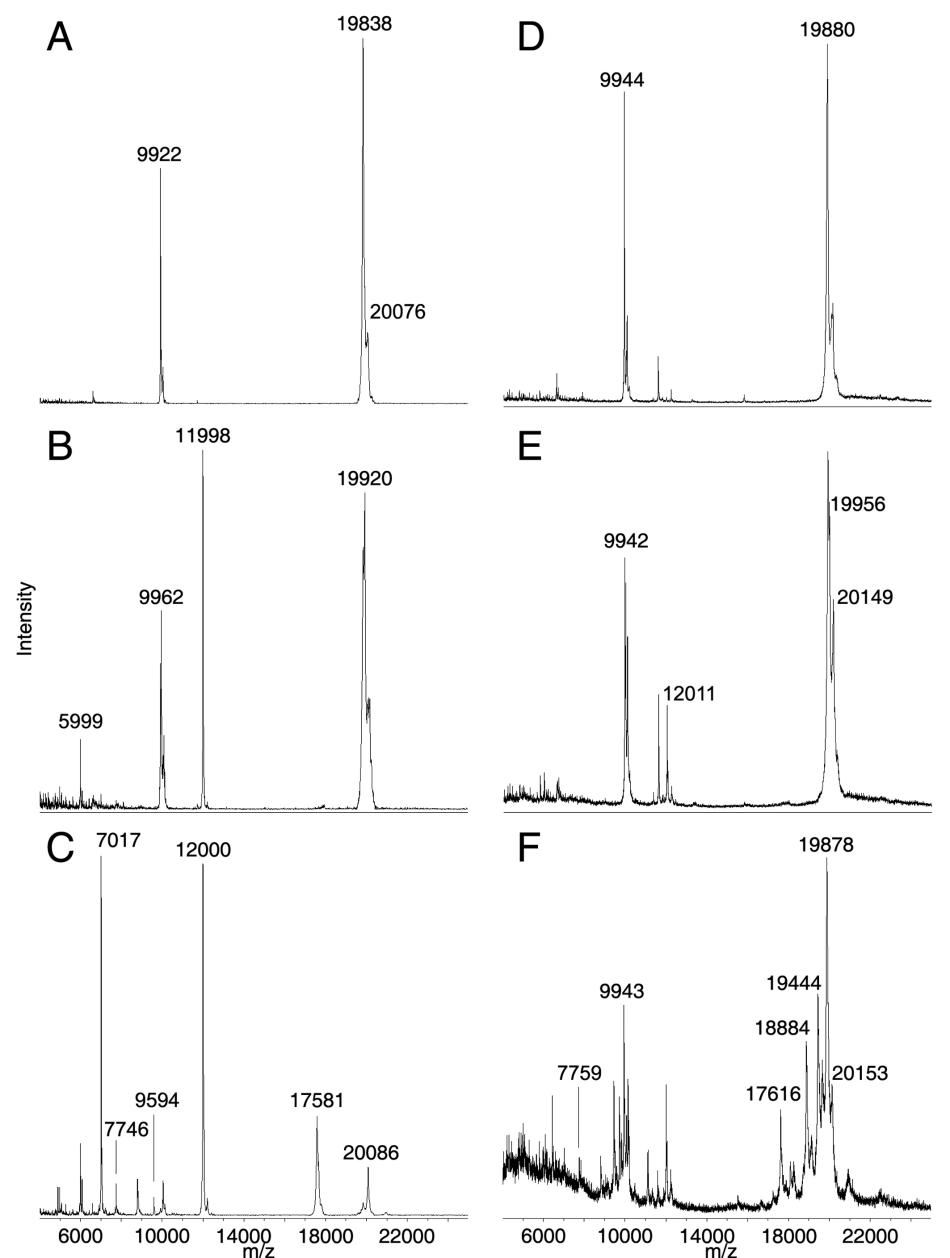
MALDI mass spectra of αA-crystallin truncation in bovine and rabbit lens. Extracted representative mass spectra from the outer cortex (**A**, **D**), middle cortex (**B**, **E**), and outer nucleus (**C**, **F**) of bovine (**A**-**C**) and rabbit (**D**-**F**) lenses are shown. Degradation products increased toward the nucleus of both species, although higher mass degradation products are more prominent in the rabbit lenses than in the bovine lenses.

Under the present experimental conditions, the most abundant ion signals observed in different regions of both bovine and rabbit lenses were for the two subunits of α-crystallin (Figure 1). In the outer and middle cortex regions, both species exhibited similar MALDI spectra. In the bovine lens, the most abundant ion signals in the outer cortex were for αA-crystallin at *m/z* 19838 and *m/z* 9922 (predicted [M+H]^+^=19833, [M+2H]^2+^=9918; Figure 1A). Similarly, the rabbit lens outer cortex exhibited little αA-crystallin degradation as ion signals for full-length αA-crystallin at *m/z* 19880 and *m/z* 9944 (predicted [M+H]^+^=19880, [M+2H]^2+^=9942) were most abundant (Figure 1D).

In the lens middle cortex, ion signals for intact αA- and αB-crystallin were again most abundant (Figure 1B,E); however, additional ion signals of approximately 80 mass units higher for αA-crystallin were also detected in both species. These signals represent phosphorylated αA-crystallin and will be discussed later. Increased ion signals that represent the αA-crystallin degradation product 1–101 in the bovine lens at *m/z* 11998 (predicted [M+H]^+^=11993) and in the rabbit lens at *m/z* 12011 (predicted [M+H]^+^=12009) were also observed.

In the bovine and rabbit lens nucleus, numerous degradation products of αA-crystallin were observed (Figure 1C,F, respectively). Interestingly, a higher number of high mass degradation products were observed in rabbit lens than in bovine lens. By acquiring MALDI mass spectra at 100 μm intervals across the tissues, it was possible to plot two-dimensional molecular images of these degradation products.

### Bovine lens α-crystallin degradation

**Figure 2:**
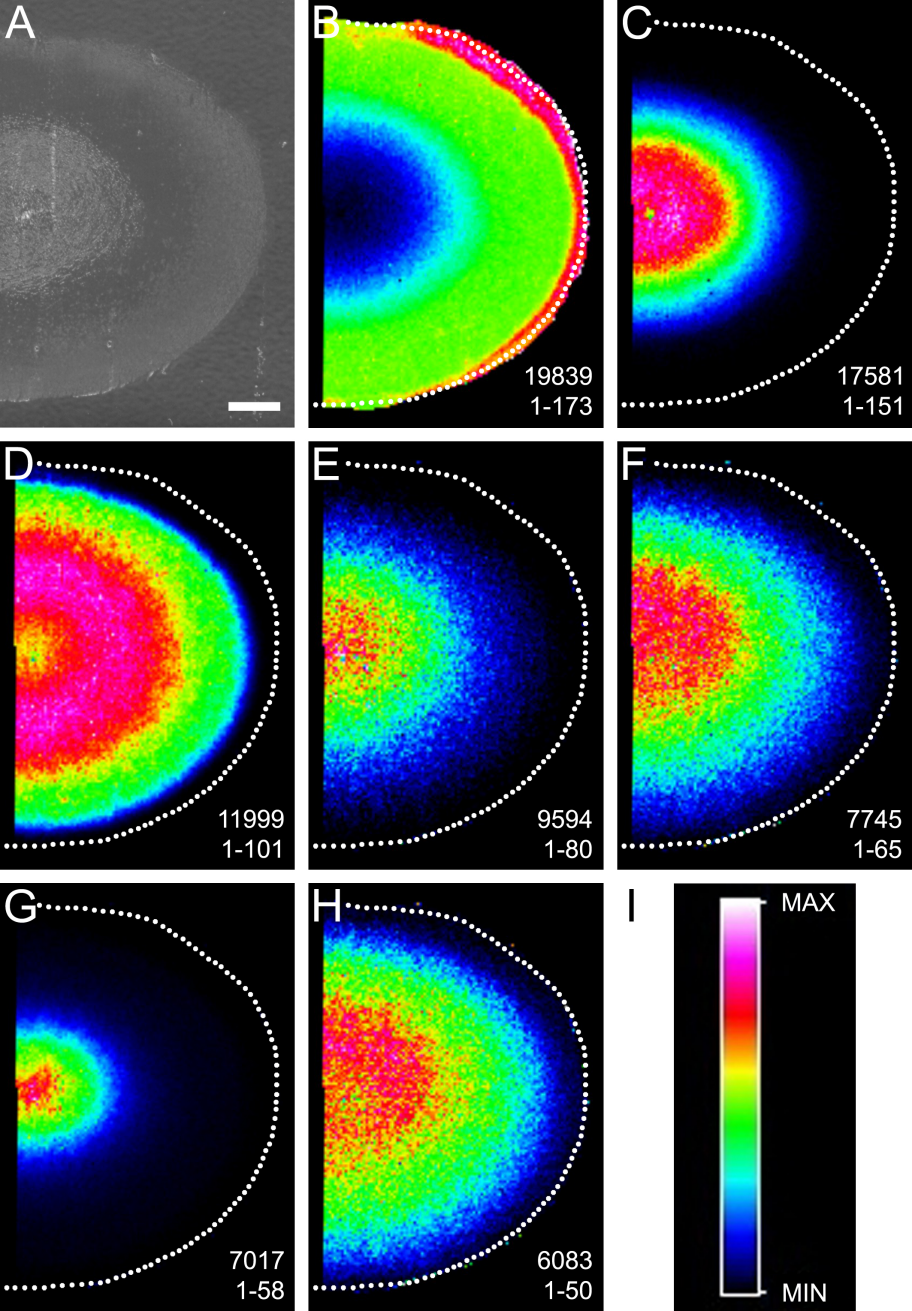
Bovine lens αA-crystallin degradation. **A **shows the optical scan of a bovine lens equatorial cryosection before MALDI matrix deposition. MALDI molecular images indicate the distribution of full-length αA-crystallin (1–173, *m/z*=19838) (**B**) and its major truncation products (**C**-**H**) in bovine lenses. The identities and observed *m/z* of these truncation products are (**C**) 1–151, *m/z*=17581, (**D**) 1–101, *m/z*=11999, (**E**) 1–80, *m/z*=9594, (**F**) 1–65, *m/z*=7745, (**G**) 1–58, *m/z*=7017, and (**H**) 1–50, *m/z*=6083. **I**: The rainbow scale was used to plot all single *m/z* molecular images. Scale bar=2 mm.

Figure 2 shows the MALDI tissue images for intact αA-crystallin and many of its detected degradation products in the bovine lens. An optical image of the equatorial tissue slice that was prepared for MALDI MS is shown in Figure 2A as reference for the following molecular images. Intact αA-crystallin was detected predominantly in the outer and middle cortex and was ostensibly absent from the lens nucleus (Figure 2B). The major degradation products of αA-crystallin showed varying degrees of complementarity with the intact form; αA-crystallin 1–101 at *m/z* 11999 (predicted [M+H]^+^=11993), 1–80 at *m/z* 9594 (predicted [M+H]^+^=9589), 1–65 at *m/z* 7745 (predicted [M+H]^+^=7741), and 1–50 at *m/z* 6083 (predicted [M+H]^+^=6080) were detected in the middle cortex and nucleus, whereas 1–151 at *m/z* 17581 (predicted [M+H]^+^=17572) and 1–58 at *m/z* 7017 (predicted [M+H]^+^=7012) were detected only in the lens nucleus. Interestingly, αA-crystallin 1–101 abundance decreased slightly in the lens nucleus possibly due to further degradation of the polypeptide to other, smaller observed degradation products. Few degradation products were detected for αB-crystallin, which is consistent with previous findings (data not shown) [21].

### Rabbit lens α-crystallin degradation

**Figure 3:**
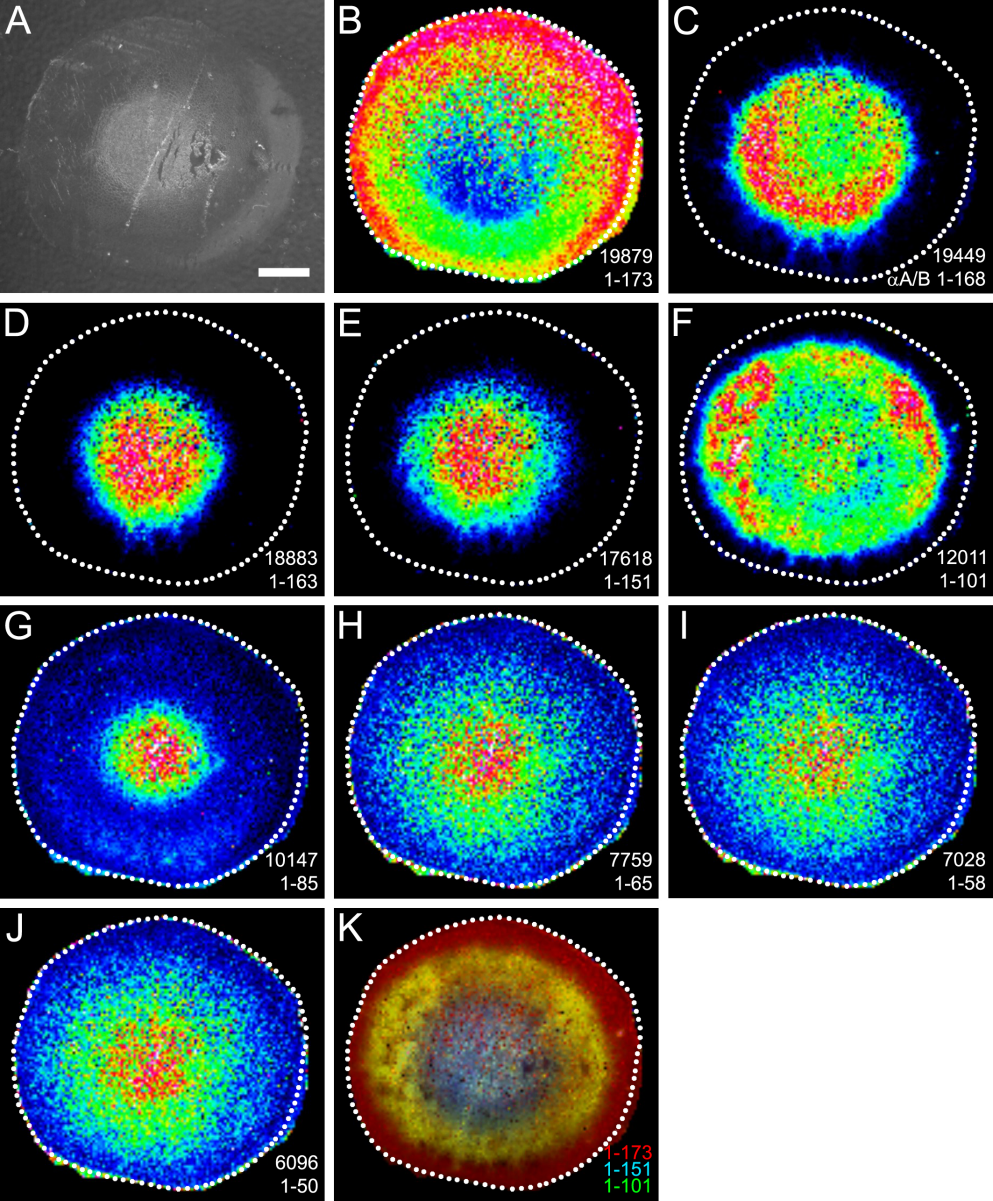
Rabbit lens αA-crystallin degradation. **A **shows the optical scan of a rabbit lens equatorial cryosection before MALDI matrix deposition. (**B**-**K**) MALDI molecular images indicate the distribution of the major forms of αA-crystallin in the rabbit lenses. The identities and observed *m/z* of these truncation products are (**B**) 1–173, *m/z*=19879, (**C**) αA or αB 1–168, *m/z*=19449, (**D**) 1–163, *m/z*=18883, (**E**) 1–151, *m/z*=17618, (**F**) 1–101, *m/z*=12011, (**G**) 1–85, *m/z*=10147, (**H**) 1–65, *m/z*=7759, (**I**) 1–58, *m/z*=7028, and (**J**) 1–50, *m/z*=6096. **K**: The molecular image shows the relationship of a full-length αA-crystallin (red) to its most abundant truncation products, 1–101 (green) and 1–151 (blue), which are found exclusively in the lens nucleus. Scale bar=2 mm.

Similar spatial distribution patterns for αA-crystallin and its major degradation products were obtained in the whole rabbit lens (Figure 3). Full-length αA-crystallin was most abundant in the outer lens cortex and was largely absent in the lens nucleus (Figure 3B). Furthermore, the major degradation products for rabbit αA-crystallin also showed a complementary distribution to full-length αA-crystallin. They increased in abundance toward the lens nucleus. Interestingly, a greater number of higher mass degradation products were detected in the rabbit lens when compared to the bovine lens. Signals for αB-crystallin 1–170 at *m/z* 19654 (predicted [M+H]^+^=19654; molecular image not shown) and αA-crystallin 1–163 at *m/z* 18883 (predicted [M+H]^+^=18880) were abundant in older lens fibers. In addition, a strong signal was detected at *m/z* 19449. This signal most likely represents either αA- or αB-crystallin 1–168. However, since the predicted molecular weights of both peptides are very similar (αA 1–168 predicted [M+H]^+^=19451, αB 1–168 predicted [M+H]^+^=19454), a definitive assignment could not be made. Additionally, the presence of both of these degraded forms of αA- and αB-crystallin in the rabbit lens cannot be ruled out. While detection of some of these degradation products in the bovine lens has previously been reported, it is likely based on this data that the degradation of major lens proteins is different in unrelated species.

Molecular images for rabbit αA-crystallin degradation products that were observed in the bovine lens were also plotted, 1–151 at *m/z* 17618 (predicted [M+H]^+^=17619), 1–101 at *m/z* 12011 (predicted [M+H]^+^=12009), 1–65 at *m/z* 7759 (predicted [M+H]^+^=7757), 1–58 at *m/z* 7028 (predicted [M+H]^+^=7028), and 1–50 at *m/z* 6096 (predicted [M+H]^+^=6096). Further contrast with αA-crystallin degradation products in the bovine lens was noted with the detection of αA-crystallin 1–85 at *m/z* 10147 (predicted [M+H]^+^=10147) in the rabbit lens.

### Relative abundance of phosphorylated α-crystallin in bovine and rabbit lenses

**Figure 4:**
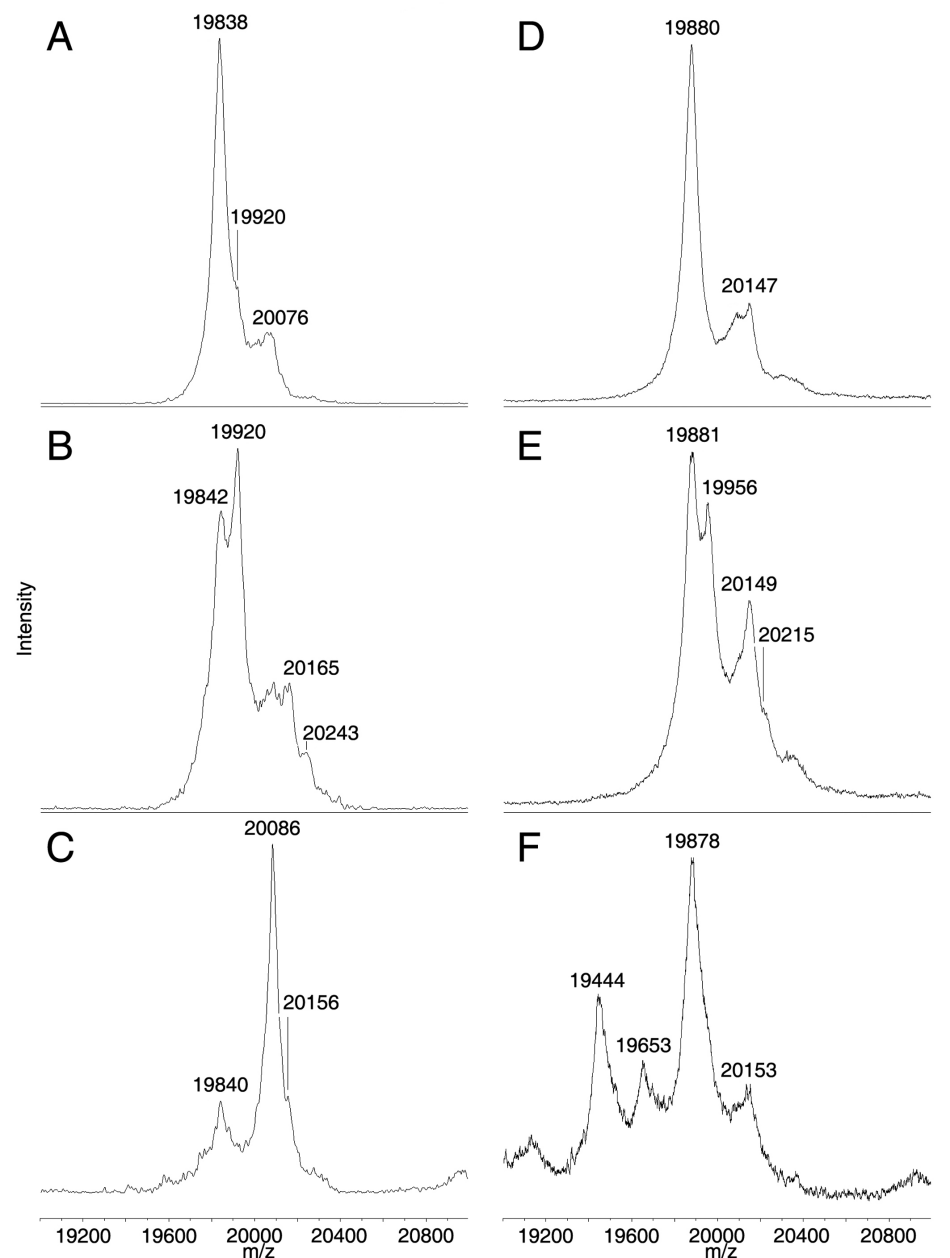
Relative abundance of phosphorylated α-crystallin in different regions of bovine and rabbit lenses. Extracted representative mass spectra of phosphorylation of intact αA- and αB-crystallin from outer cortex (**A**, **D**), middle cortex (**B**, **E**), and outer nucleus (**C**, **F**) of bovine (**A**-**C**) and rabbit (**D**-**F**) lenses are shown. Phosphorylation of both subunits of α-crystallin is most abundant in the middle cortex of bovine and rabbit lenses.

In the lens, phosphorylation of both subunits of α-crystallin has been shown to occur [22-24]. Signals for unmodified intact α-crystallin subunits and signal shifts of +80 mass units from the predicted masses for αA- and αB-crystallin are observed in extracted MALDI mass spectra from different regions of bovine and rabbit lenses (Figure 4). These shifted signals are assigned to the singly-phosphorylated forms of intact αA- and αB-crystallin. Phosphorylated bovine αA-crystallin at *m/z* 19920 and *m/z* 9962 (predicted [M+H]^+^=19914, [M+2H]^2+^=9959) and phosphorylated rabbit αA-crystallin at *m/z* 19956 and *m/z* 9982 (predicted [M+H]^+^=19960, [M+2H]^2+^=9982) were observed in higher abundance in the middle cortex (Figure 4B,E) than in the outer lens cortex (Figure 4A,D). Signals assigned to singly-phosphorylated αB-crystallin were also observed in bovine lenses at *m/z* 20165 (predicted [M+H]^+^=20161) and rabbit at *m/z* 20215 (predicted [M+H]^+^=20230). In addition, a signal shift of +160 mass units from unmodified bovine αB-crystallin was also observed predominantly in the lens middle cortex and was assigned to doubly-phosphorylated αB-crystallin at *m/z* 20243 (predicted [M+H]^+^=20241). Signals for all phosphorylated forms of α-crystallin decreased in the lens outer nucleus in both species (Figure 4C,F).

### Bovine lens α-crystallin phosphorylation

**Figure 5:**
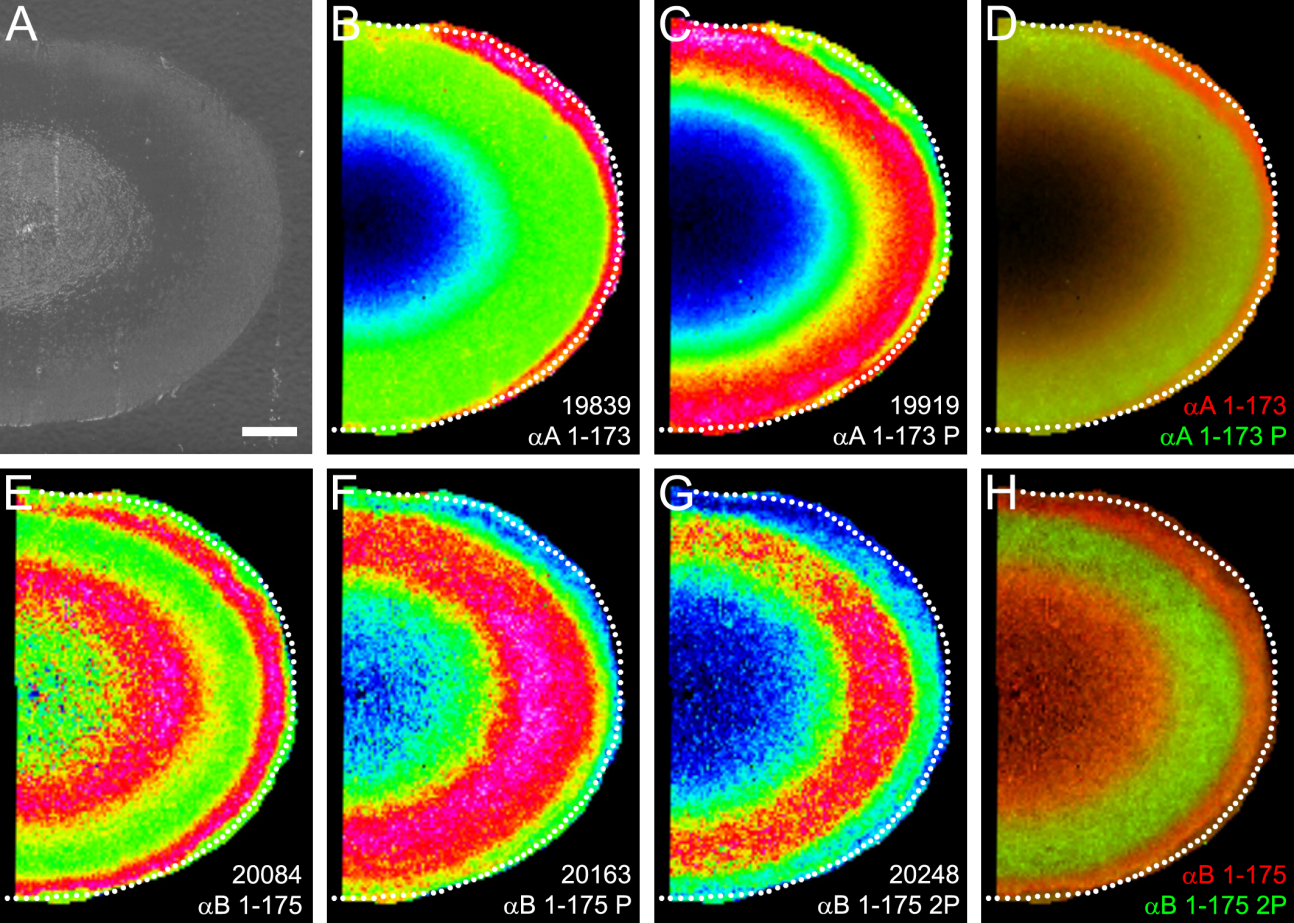
Bovine lens α-crystallin phosphorylation. **A **shows the optical scan of a bovine lens equatorial cryosection before MALDI matrix deposition. **B** illustrates the distribution of full-length αA-crystallin. **C**: Singly-phosphorylated full-length αA-crystallin (observed *m/z*=19920) shows higher abundance in the middle cortex. **D**: The dual color image shows the relationship between the spatial distributions of full-length αA-crystallin (red) and phosphorylated αA-crystallin (green). **E**: The distribution of full-length αB-crystallin (residues 1–175, observed *m/z*=20084) showed higher abundance toward the edge of the lens and in the inner cortex. Singly- phosphorylated (**F**) and doubly- phosphorylated (**G**) αB-crystallin (observed *m/z*=20163 and 20248, respectively) are more abundant in the middle cortex. **H**: The dual color image shows the relationship between the spatial distributions of full-length αB-crystallin (red) and doubly-phosphorylated αB-crystallin (green). P/2P=phosphorylated forms of protein. Scale bar=2 mm.

The molecular images of unphosphorylated and phosphorylated forms of bovine α-crystallin are presented in Figure 5. Unphosphorylated αA-crystallin was most abundant in a narrow zone at the edge of equatorial lens sections (Figure 5B) while phosphorylated αA-crystallin at *m/z* 19920 and *m/z* 9962 (predicted [M+H]^+^=19914, [M+2H]^2+^=9959) was most abundant in the lens middle cortex (Figure 5C). Both signals decreased dramatically in the lens nucleus. A dual-color overlay of unphosphorylated (red) and phosphorylated (green) αA-crystallin is presented in Figure 5D. In contrast, unphosphorylated intact αB-crystallin was more ubiquitous in the lens but was most abundant toward the edge and in the outer nucleus (Figure 5E). Both singly- phosphorylated αB-crystallin at *m/z* 20165 (predicted [M+H]^+^=20161) and doubly- phosphorylated αB-crystallin at *m/z* 20243 (predicted [M+H]^+^=20241) were most abundant in the lens middle cortex and decreased dramatically in the lens nucleus (Figure 5F,G, respectively). Furthermore, the increase in the abundance of phosphorylated αB-crystallin coincided with a relative decrease in unphosphorylated αB-crystallin in the lens middle cortex. A dual-color overlay of unphosphorylated (red) and doubly-phosphorylated (green) αB-crystallin indicates the complementarity of the spatial distribution of phosphorylated and unphosphorylated αB-crystallin (Figure 5H).

The above findings are consistent with previous MALDI imaging of bovine α-crystallin but offer greatly improved spatial resolution (100 μm step-size compared to 250 μm step-size) that confirmed the presence of phosphorylation rings of both αA- and αB-crystallin in the lens middle cortex.

### Rabbit lens α-crystallin phosphorylation

**Figure 6:**
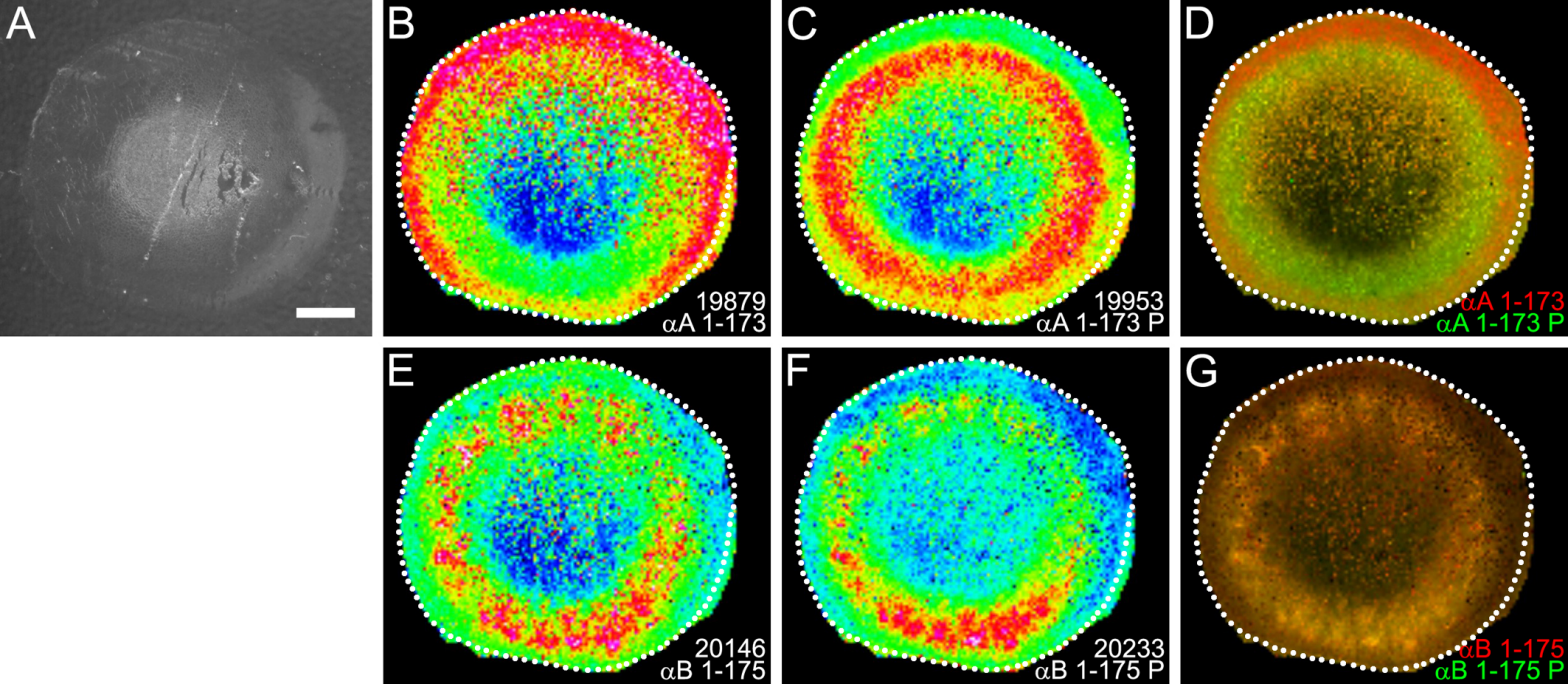
Rabbit lens α-crystallin phosphorylation. **A** shows the optical scan of a rabbit lens equatorial cryosection before MALDI matrix deposition. **B** shows the distribution of full-length αA-crystallin. **C**: Singly-phosphorylated full-length αA-crystallin (observed *m/z*=19953) shows higher abundance in the middle cortex. **D**: The dual color image shows the relationship between the spatial distributions of full-length αA-crystallin (red) and phosphorylated αA-crystallin (green). Both full-length αB-crystallin (**E**; residues 1–175, observed *m/z*=20146) and the singly-phosphorylated αB-crystallin (**F**; observed *m/z*=20215) show higher abundance in the middle cortex. **G**: The dual color image shows the relationship between the spatial distributions of full-length αB-crystallin (red) and phosphorylated αB-crystallin (green). Yellow coloration indicates colocalization. P=phosphorylated form of protein. Scale bar=2 mm.

The distributions of unphosphorylated and phosphorylated α-crystallin in the rabbit lens are presented in Figure 6. Like bovine αA-crystallin, unphosphorylated rabbit αA-crystallin was most abundant at the edge of the lens (Figure 6B) while phosphorylated rabbit αA-crystallin at *m/z* 19956 and *m/z* 9982 (predicted [M+H]^+^=19960, [M+2H]^2+^=9982) was most abundant in the lens middle cortex (Figure 6C). Both of these signals were less abundant in the lens nucleus. The dual-color image of these two signals confirms their different spatial distributions (Figure 6D). However, in contrast to unphosphorylated bovine αB-crystallin distribution, both unphosphorylated and phosphorylated rabbit αB-crystallin at *m/z* 20215 (predicted [M+H]^+^=20230) were most abundant in the lens middle cortex (Figure 6E,F, respectively). The dual-color image of unphosphorylated (red) and singly-phosphorylated (green) α-crystallin confirms areas of colocalization (yellow) of these protein forms in the lens middle cortex. Doubly-phosphorylated αB-crystallin was not detected in the rabbit lens.

## Discussion

The posttranslational modification of existing lens proteins is a mechanism by which differentiated lens fiber cells adapt to a changing cellular environment [6]. MALDI imaging mass spectrometry is an ideal tool to study the spatial distribution of posttranslational modifications of the lens protein, α-crystallin. Standard immunohistochemical approaches that are often used to study protein distributions would be challenged by the lack of specific antibodies to phosphorylated forms, the inability to detect specific truncation products, and the large number of individual experiments required to obtain this information. While MALDI imaging mass spectrometry eliminates more complex protein extraction and separation techniques often used in other proteomic studies, tissue preparation is nevertheless crucial to the acquisition of high quality data.

A new tissue preparation procedure for MALDI imaging of lens α-crystallin has been developed, and its utility has been demonstrated by applying the procedure to study the spatial distribution of bovine and rabbit lens α-crystallin. The detection of similar distributions of truncated αA-crystallin in the bovine lens to previous results validates the current tissue preparation method [21]. Furthermore, due to improved spatial resolution, the predominance of phosphorylated αA- and αB-crystallin in the lens middle cortex is confirmed. In comparing the bovine and rabbit lens α-crystallin images, similar spatial distributions of unmodified and modified α-crystallin were observed in both species in general, however, some significant differences were noted. A greater abundance of higher mass/longer α-crystallin truncation products was observed in the rabbit lens. In contrast to αB-crystallin distribution in the bovine lens, intact rabbit lens αB-crystallin was most abundant in the lens middle cortex. These observations highlight differential expression and processing of a highly conserved protein in two different species.

The current tissue preparation method worked well in both bovine and rabbit lenses; however, higher quality mass spectral data were obtained in the bovine lens especially toward the core region (see Figure 1). While the precise reason for this is not known, the composition of lipids may be a contributing factor. Lipids are generally good target analytes for MALDI analysis since they ionize easily. However, their presence in tissue sections may interfere with matrix/protein co-crystallization, hence compromising protein ionization efficiency in the mass spectrometer. Previously, the composition of lens phospholipids has been shown to differ between species [25] and in different regions of the lens [26]. In addition, while the tissue preparation procedure for MALDI imaging included several washes aimed at tissue dehydration, fixation, and removal of physiological salts and phospholipids, it is possible that not all phospholipids were removed by this procedure, hence contributing to the variability in mass spectral data quality between species. Nevertheless, the current tissue preparation procedure and matrix/solvent system serves as a robust starting point for the application and optimization of MALDI imaging techniques to lenses from different species. The results presented in the current study indicate that tissue preparation methods must be optimized not only for each tissue but also for equivalent tissue in different species.

Age-dependent truncation of lens αA-crystallin is a well known and well characterized phenomenon. Nonenzymatic cleavage of bovine αA-crystallin has been shown to occur at Asn-101 [27] while calpain-mediated cleavage at Asp-151, Glu-156, Arg-163, and Ser-168 occurs in the rat lens [28]. The abundance of bovine αA-crystallin 1–101, which was highest in the lens outer nucleus, decreased in the central lens nucleus and has not previously been reported. This spatial distribution may represent the further breakdown of αA-crystallin 1–101 to lower molecular weight forms of the protein, which were observed in highest abundance in the central lens nucleus. This phenomenon may also be reflected in the spatial distribution patterns of rabbit αA/αB-crystallin 1–168 and αA-crystallin 1–101, which is also most abundant in the lens outer nucleus. Differences in the truncation products between bovine and rabbit lens αA-crystallin were observed with more abundant higher mass truncation products (αA/αB 1–168 and αA 1–163) observed in the rabbit lens (see Figure 3C,D). Since αA-crystallin is highly conserved between bovine and rabbit at all detected truncated residues, the observed differences in truncation products are not due to sequence differences. Additionally, these differences are unlikely to be due to differences in lens age since both lenses were approximately two years old. Instead, it is possible that each species exhibits either differential expression or activity of lens enzymes such as calpains, which may contribute to increased formation of higher mass αA-crystallin truncation products observed in the rabbit lens. Despite the observed species differences in αA-crystallin truncation products, COOH-terminal truncation of αA-crystallin is known to decrease its chaperone activity [11-13], which is thought to promote increased protein aggregation in the center of the lens and the formation of lens opacities. Furthermore, the abundance and diversity of truncated α-crystallin forms must be considered in the context of lens physiology and lens aging. For example, some products may function as mini-chaperones [29] while others may be unstable and proaggregatory.

It is noteworthy that well known lens protein modifications in the nucleus region such as aggregation/insolubilization and protein–protein cross-linking would likely reduce the mass spectral signals for α-crystallin subunits. It is possible that based on the analysis of intact αA-crystallin distributions alone, this effect could lead to an overrepresentation of αA-crystallin truncation in the lens nucleus. However, since the decreased signal for intact αA-crystallin in the lens nucleus (Figure 2B) is accompanied by an increase in its truncation products (Figure 2C-H) and the intensity of bovine αB-crystallin is similar in the outer cortical and nuclear regions (Figure 5E), this suggests that the disappearance of the αA-crystallin signal in the bovine and rabbit lens nucleus is not substantially affected by aggregation/insolubilization and cross-linking.

A striking feature of α-crystallin distribution in both species is the zone of α-crystallin phosphorylation in the lens middle cortex. Phosphorylation is a common form of posttranslational modification known to alter the function of proteins. Such a posttranslational modification is particularly significant in the setting of lens fiber cells, which lose the ability to synthesize new protein as they age and must find other ways to modify protein function to adapt to the changing lens fiber cell environment. Both subunits of α-crystallin are phosphorylated by cAMP-dependent kinase in bovine lens extracts [30-32], αA-crystallin at Ser-122 [24] and αB-crystallin at Ser-19, Ser-45, and Ser-59 [22,23]. Additionally, a lens phosphatase has been shown to dephosphorylate both subunits [33], suggesting that in the lens, α-crystallin phosphorylation may be reversible. This suggestion is consistent with our MALDI imaging results, particularly for bovine αB-crystallin, where rings of unphosphorylated αB-crystallin were observed both in the lens outer cortex and outer nucleus while singly- and doubly-phosphorylated αB-crystallin was most abundant in the lens middle cortex (see Figure 5E-H). Interestingly, doubly-phosphorylated αB-crystallin was not detected in the rabbit lens. This contrast between bovine and rabbit lenses is not due to sequence differences as the known phosphorylated residues in bovine αB-crystallin are conserved in the rabbit. Furthermore, since the age of each lens is approximately two years, it is less likely that the observed differences in αB-crystallin phosphorylation are due to age differences. Instead, this difference may be due to differential protein processing between species, which has already been shown through the observation of different degradation products for α-crystallin between the bovine and rabbit (see Figure 1).

In the lens, phosphorylation of α-crystallin has been associated with numerous biological processes including protein translocation [34,35] and interaction with cytoskeletal proteins [36-40], which may be involved in morphological remodeling of differentiating lens fiber cells. In addition, the studies of αB-crystallin phosphorylation mimics indicate that phosphorylation of this subunit alters substrate binding and subsequent molecular chaperone activity [15,17]. Therefore, phosphorylation is an important functional regulator of this α-crystallin protein subunit. However, information pertaining to the spatial localization of phosphorylated α-crystallin in the lens has been largely limited to electrophoretic analysis of microdissected lens regions. These studies have indicated that both α-crystallin truncation and other forms of α-crystallin modification increase with increasing cell age and depth in the lens, although the nature of these modifications have been unclear [19,20]. In addition, the spatial resolution of these studies is limited by the microdissection technique.

In summary, a new method has been developed to map the distribution of α-crystallin and its most abundant modified forms in the ocular lens by MALDI imaging mass spectrometry. While this general method worked well in both bovine and rabbit lenses, the present results suggest that species-dependent tissue preparation is required to maximize the information obtained in each MALDI imaging mass spectrometry experiment. Moreover, the current study indicates that consistent with previous reports, α-crystallin modification increases with cell age and depth in the lens. Lastly, based on the MALDI imaging results of lens α-crystallin, the middle cortex is an important region in the lens where a variety of functional changes related to protein phosphorylation and truncation may take place simultaneously. However, the precise nature of those changes, the signals for α-crystallin phosphorylation and dephosphorylation (spatial/biochemical), and their effect on global lens function remain unknown and require further investigation.
